# Evaluation of the Effects of Radiation Therapy on Muscle Contractibility and Skin Healing: An Experimental Study of the Cancer Treatment Implications

**DOI:** 10.3390/life13091838

**Published:** 2023-08-30

**Authors:** Sarah O. M. Avelino, Rafael M. Neves, Leonardo A. Sobral-Silva, Rubens N. Tango, Claudio A. Federico, Mariana R. C. Vegian, Luis Augusto de Almeida-Silva, Estela Kaminagakura, José Benedito O. Amorim, Luana M. R. Vasconcellos

**Affiliations:** 1 Department of Bioscience and Oral Diagnosis, Institute of Science and Technology, São Paulo State University (UNESP), Avenida Engenheiro Francisco José Longo, 777, Jardim São Dimas, São José dos Campos 12245-001, SP, Brazil; 2 Department of Dental Materials and Prosthodontics, Institute of Science and Technology, São Paulo State University (UNESP), Avenida Engenheiro Francisco José Longo, 777, Jardim São Dimas, São José dos Campos 12245-001, SP, Brazil; 3 Department of Aerospace Science and Technology, Institute of Advanced Studies, Technological Institute of Aeronautics (ITA), Praça Marechal Eduardo Gomes, 50, Vila das Acacias, São José dos Campos 12228-615, SP, Brazil

**Keywords:** radiotherapy, muscle, skin, electromyography, in vivo

## Abstract

Background: Radiotherapy can affect healthy cells, resulting in side effects. This study aimed to assess the impact of radiotherapy on soft tissue in surgical wounds in rats. Methods: The animals were divided into four groups: control (S) group without irradiation, immediate irradiation (S-IIr) group receiving irradiation right after surgery, late irradiation (S-LIr) group receiving irradiation four weeks after surgery, and early irradiation (Ir-S) group receiving irradiation before surgery. The irradiated groups underwent two fractional stages of 15 Gy. Muscle contractibility (EMG) was evaluated at two different time points, and after 2 and 7 weeks, the animals were euthanized for histological analysis of the muscles and skin. Results: There was no significant difference between the EMG1 and EMG2 values of the S and S-LIr groups, but both S-IIr and Ir-S groups exhibited a statistically significant difference. The S group demonstrated a larger diameter of muscle fiber compared to other groups, showing a significant difference. In terms of skin analysis, the S-IIr group had the least inflammatory infiltrate and the highest amount of red fibers, differing significantly from the other groups. Conclusions: Regardless of the duration, radiotherapy was found to have effects on the surrounding soft tissues, as concluded by this study.

## 1. Introduction

The treatment of malignant tumors of the head and neck has been carried out through combinations of techniques and multidisciplinary care involving surgery, radiotherapy, chemotherapy, medical, dental, speech therapy, and nutritional and psychological assistance [1]. Radiotherapy has been used for over 100 years in cancer treatment. However, the radiation used to combat malignancy is unfortunately not selective for neoplastic cells and can also damage the healthy tissue surrounding the cancer [2].

In the field of dentistry, the rehabilitation of patients who have undergone cancer treatment with radiotherapy becomes a challenge due to the side effects of ionizing radiation in the tissues. The irradiation absorbed by the tissues can generate several undesirable side effects and negative influences on the surrounding soft tissues, including oral mucositis, xerostomia, fibrosis of the blood vessels and soft tissues, and trismus [3,4,5].

Although radiotherapy is an effective treatment for the reduction or elimination of head and neck cancer, it presents severe toxicities to the structures and tissues adjacent to the tumor, such as the muscles and skin [6]. Trismus is the condition in which the patient’s mouth opening ability is limited and can result when masticatory muscles are part of the area covered by irradiation [7,8,9]. This limitation of mouth opening has a great impact on the life quality of individuals because, in addition to affecting mandibular mobility, it compromises both oral hygiene and other dental care [10,11,12]. Such impairments can serve as triggers for even more severe complications, such as osteoradionecrosis [13].

Changes in the skin were also observed, including atrophy and inflammation [5,14], erythema, flaking, blistering, ulcerations, necrosis, pain, and burning [11]; such changes are commonly called radiodermatitis [15].

The present study, using the experimental model of surgical procedures in rats, evaluated the effects of irradiation on muscle contractability as well as skin epithelial tissue. This study contributes to research that evaluates the side effects of radiotherapy in soft tissues by simulating patients who have received radiation therapy at different times, before and after the surgical procedure.

## 2. Materials and Methods

This project was approved by the Animal Ethics Committee (CEUA, Protocol 003/2016) of the Institute of Science and Technology of the Campus of São Jose dos Cam-pos/UNESP and was carried out in accordance with the ethical principles adopted by the Brazilian National Animal Care Ethical Council (CONCEA).

Twenty-four Wistar rats, 90 days old and weighing about 300 g each, were used in this study. Throughout the experiment, the animals remained in a carefully monitored environment maintained at a temperature of around 20 °C and a humidity of 55%, and they received water and feed ad libitum. After 30 days of adaptation to the environment, with daily cycles alternating every 12 h corresponding to the day and night periods, this experiment was started. We followed a randomized, prospective, controlled animal model design in accordance with all the recommendations of the “Animal Research: Reporting In Vivo Experiments” (ARRIVE) [16] guidelines for the execution and submission of studies on animals.

### 2.1. Experimental Setup

**Figure 1:**
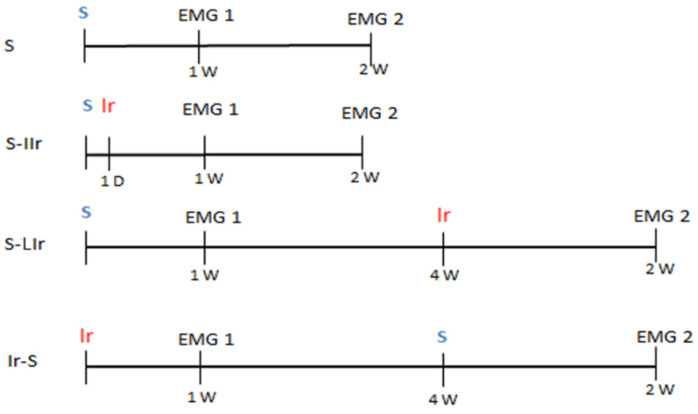
Experimental design timeline of surgical wound, irradiation, and electromyography for different groups.

The animals were randomly divided according to the following treatment groups (Figure 1): (a) control group: surgical wound without irradiation (S); (b) immediate irradiation group (S-IIr): surgical wound followed by irradiation after 1 day; (c) late irradiation group (S-LIr): surgical wound and irradiation after 4 weeks; (d) early irradiation group (Ir-S): irradiation and, after 4 weeks, surgical wound.

The S-IIr and S-LIr treatments had the objective of simulating the clinical situation in which a patient undergoes radiotherapy sessions 1 month and 2 years after the surgical wound, respectively. Regarding the Ir-S group, the objective was to simulate the clinical situation in which the patient was submitted to a surgical procedure 2 years after the conclusion of radiotherapy treatment. All periods of irradiation and euthanasia were determined based on a previous study that correlated the age of adult rats and humans [17].

### 2.2. Surgical Procedure to Surgical Wound

For the surgical procedures, the animals were anesthetized with xylazine hydrochloride (Anasedan-Vetbrands, Jacareí, SP, Brazil) and ketamine (Dopalen-Agibrands Brasil Ltd., Paulínia, SP, Brazil). Then, the surgical sites of the right femur were submitted to trichotomy and antisepsis with an iodized alcohol solution, and incisions were performed in the region corresponding to the medial face of these femurs. The muscles were divulsed to simulate the clinical situation of the surgical wound, and the tissues were repositioned and sutured with silk thread #4 (Ethicon/Johnson & Johnson, São José dos Campos, SP, Brazil). Antisepsis was performed again with iodinated alcohol. After the procedures, all animals received a subcutaneous injection with 0.1 mg/kg of buprenorphine every 12 h for 3 days for pain relief. The animals were housed in cages and monitored until the euthanasia period of 2 weeks.

### 2.3. Irradiation Procedure

In order to perform the radiation procedure, the animals were anesthetized for surgery and immobilized in a standardized manner using equipment developed especially for this study. The immobilization was conducted under an Eldorado model 76 tele-thermotherapy irradiator from Atomic Energy of Canada Limited (Chalk River, ON, Canada), with a Co gamma radiation source, located at the Institute of Advanced Studies (IEAv) of the Department of Aerospace Science and Technology (DCTA), in São José dos Campos, São Paulo. Afterwards, the animals were irradiated locally in their femurs, simulating a maximum dose radiotherapy treatment of a neoplastic lesion. Radiations of 30 Gy were divided into two procedures of approximately 15 Gy each, with sessions of approximately 1 h and 18 min duration, performed at 24 h intervals. The distance between the source and the irradiation sites was 0.9 m. The doses were defined based on the animal model used and on the tissues affected [18].

### 2.4. Analysis of Muscle Contractibility

The property of muscle contractibility in the femur region was evaluated using the EMG-800C model electromyograph (EGM System do Brasil Ltd.a, São José dos Campos, SP, Brazil). For the measurement of muscle contractibility, the animals were immobilized in a standardized manner, using equipment specially developed for this study of the muscle contraction analysis on both occasions. The first evaluation was performed one week after surgery for the S, S-IIr, and S-LIr groups, and one week after the irradiation for the Ir-S group. The second evaluation was performed prior to euthanasia in order to measure the influence of radiotherapy on muscle activity. 

### 2.5. Analysis of Soft Tissues

After the euthanasia of the animals, fixation with 10% formaldehyde was performed on soft tissue samples located in the area of the surgical wound for a minimum period of 48 h. Muscle tissue samples were separated from the skin, and both pieces were infiltrated and embedded in paraffin in separate blocks, and they were then semi-serially sectioned (5 µm) in transversal and longitudinal directions for muscle and skin, respectively.

For muscle tissue, conventional histological processing was carried out using a semi-serial process, and the sections of muscle tissue were stained with Masson’s trichrome to measure the amount of collagen fibers present among muscle fibers; a procedure that indicates the presence of fibrosis. To perform this analysis, 3 random fields were selected on the scanned sections, containing at least 4 bundles of muscle fibers. The fiber perimeters were analyzed using the Las Phases program (Leica, Wetzlar, Germany).

Histological sections of the skin were stained with hematoxylin and eosin to assess the inflammatory infiltrate and the number of blood vessels present. The inflammatory infiltrate was assessed by means of its intensity, which was related to the average number of cells present in the field. Five fields of each section were analyzed at the original 40x magnification, and the analyses were performed by a single, calibrated, blind examiner. The intensity of the inflammatory infiltrate was given in scores from 1 to 4, with Score 0: absence; Score 1: 0 to 25% of inflammatory cells; Score 2: 25 to 50% of inflammatory cells; Score 3: 50 to 75% of inflammatory cells; Score 4: more than 75% of inflammatory cells. The skin sections were also stained with Picrosirius in order to assess fiber density, which is indicative of cell renewal. After obtaining images using Pannoramic Scan equipment (3D HISTECH, Budapest, Hungary), 5 fields from each section were selected to calculate the percentage of green and red fibers present.

### 2.6. Statistical Analysis

Intra-group muscle contractability was compared using the Student’s *t* test. Then, the comparison between the groups was performed on two different occasions using the one-way ANOVA statistical test and the Tukey test (5%).

After obtaining data for the muscle fiber perimeters, inflammatory infiltrate, number of blood vessels present, and picrosirius staining, the obtained values were submitted to descriptive statistics, ANOVA One-Way statistical test, and Tukey test (5%).

All data obtained were submitted to statistical analysis using the GraphPad Prisma 7.01 software.

## 3. Results

### 3.1. Analysis of Muscular Contractibility

Muscle contractability in the animals’ femur region was measured on two occasions (one week after the surgical wound and prior to euthanasia) using the EMG-800C electromyograph (EMG System do Brasil Ltda., São José dos Campos, SP, Brazil).

**Figure 2:**
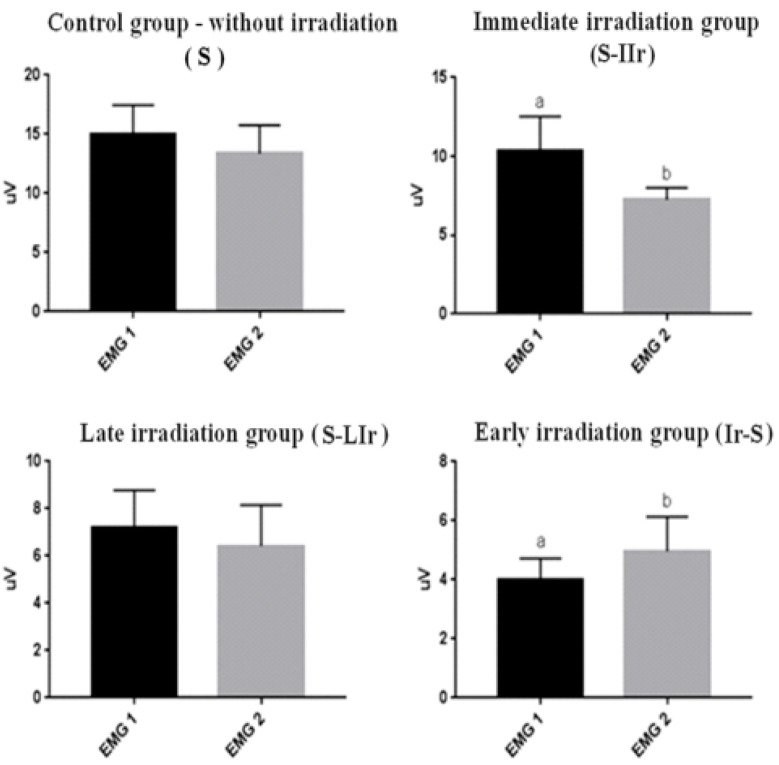
Graph of mean and standard deviation (±) of the EMG1 and EMG2 values in intragroup analysis. Distinct letters indicate statistical difference.

Figure 2 shows the comparison between EMG 1 and EMG 2 for each group; in the S and S-LIr groups, no statistical difference was observed between the contractability values obtained in the two events. However, in the S-IIr group, higher values were observed in EMG 1 than in EMG 2, with a statistical difference between the events (*p* < 0.05). On the other hand, in the Ir-S group, lower values were noted in EMG 1 than in EMG 2, with a statistical difference between the two events (*p* < 0.05).

**Figure 3:**
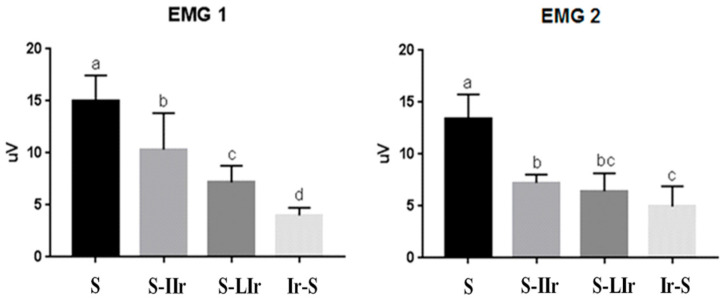
Graph of mean and standard deviation (±) of the EMG1 and EMG2 values in intergroup analysis. Distinct letters indicate statistical difference, while similar letters do not.

Afterward, the EMG values were compared intergroup for the two events. The data obtained for the EMG 1 group (Figure 3) showed that all groups differed (*p* < 0.05), with the S group exhibiting the highest capacity for muscle contractability, followed by the S-IIr, S-LIr, and Ir-S groups.

In the EMG 2 measurement (Figure 3), there was a difference between the groups; the S group continued to present the highest contractibility and differed from the other groups (*p* < 0.05). The Ir-S group again exhibited the lowest value of muscle contractability and differed statistically from the S-IIr and S groups (*p* > 0.05).

### 3.2. Histological Analysis of Muscle Tissue

**Figure 4:**
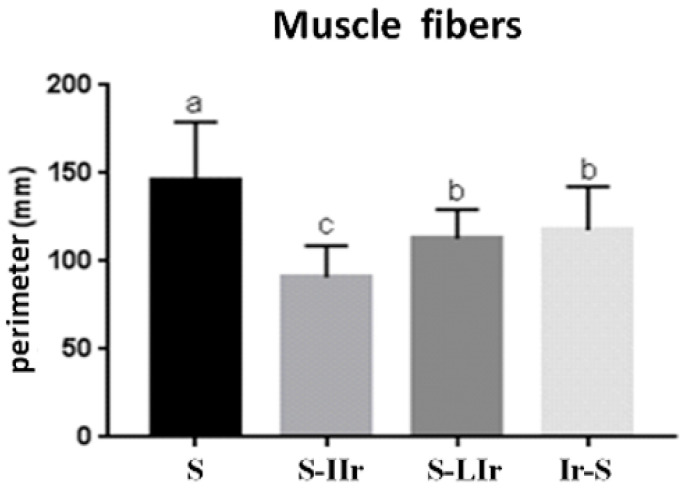
Graph of mean and standard deviation (±) of the perimeter of muscle fibers present in the different groups. Distinct letters indicate statistical difference, while similar letters do not.

The results for the influence of radiotherapy on the perimeter of muscle fibers are presented in Figure 4. The S group showed the largest perimeter of muscle fibers and differed statistically from all other groups (*p* < 0.05), while the S-IIr group had the smallest perimeter and also differed statistically from all other groups (*p* < 0.05). The S-LIr and Ir-S groups did not show any statistical difference between them (*p* > 0.05).

### 3.3. Histological Analysis of the Skin

**Figure 5:**
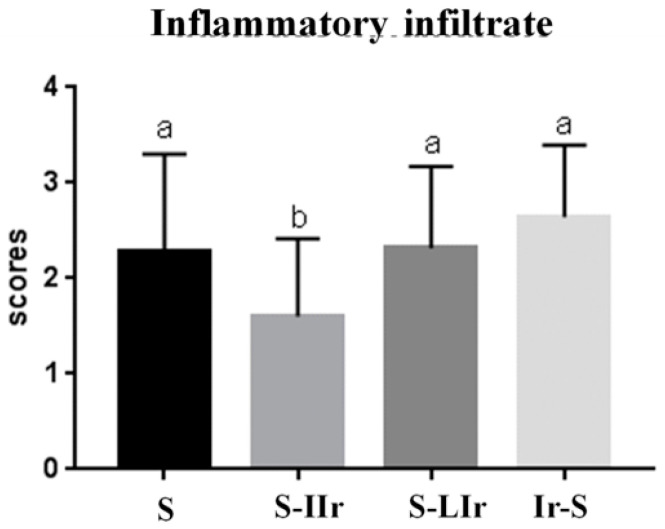
Graph of mean and standard deviation (±) of inflammatory infiltrate present in the different groups. Distinct letters indicate statistical difference, while similar letters do not.

After the statistical test, it was observed that the S-IIr group exhibited the lowest amount of inflammatory infiltrate, with a statistical difference in relation to the other groups (*p* < 0.05), among which there was no difference (*p* > 0.05) (see Figure 5).

**Figure 6:**
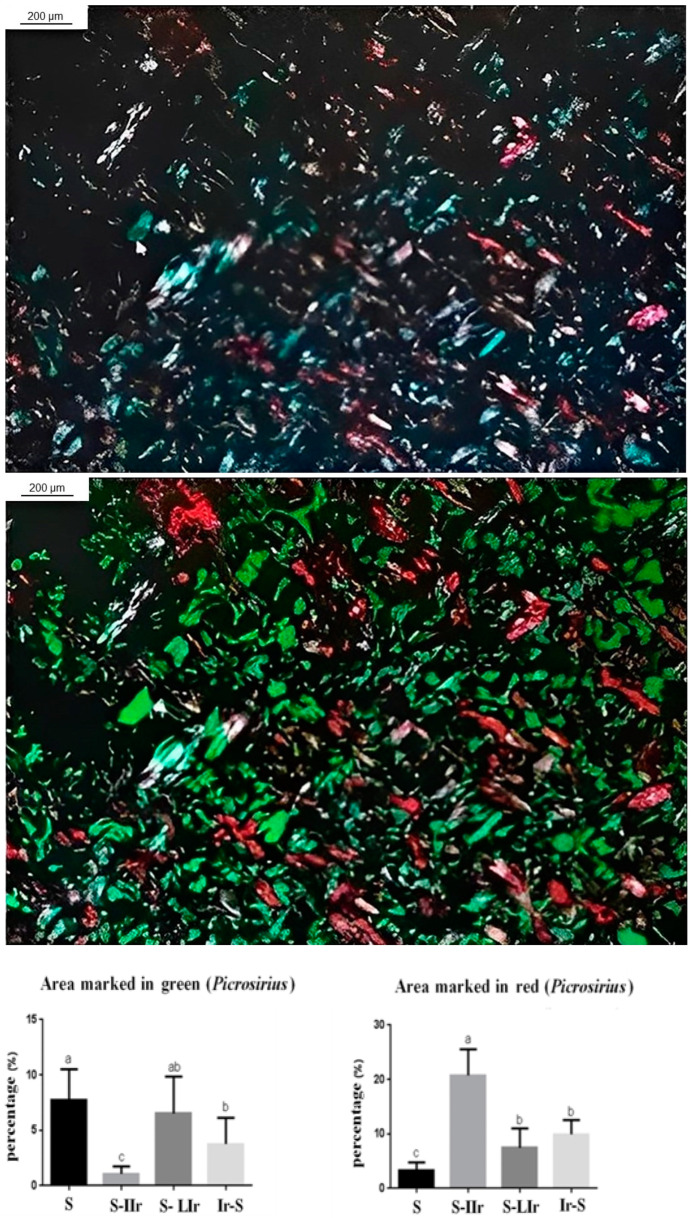
Graph of mean and standard deviation (±) of the percentage of density of collagen fibers stained by picrosirius (fibers stained green and red, respectively). Distinct letters indicate statistical difference, while similar letters do not.

For evaluation of fiber density, histological sections of the skin were also stained with Picrosirius Red (Figure 6), and it was then observed that the S-IIr group exhibited the lowest amount of fibers stained in green, with a statistical difference in relation to the other groups (*p* < 0.05). On the other hand, the S group exhibited the highest amount of green fibers, not differing statistically only from the S-LIr group (*p* > 0.05).

Regarding the fibers marked in red, the S-IIr group also exhibited a different behavior from the other groups, with the highest amount of red fibers, and showed a statistical difference (*p* < 0.05). The S group had the lowest amount of red fibers, which also differed from the other groups (*p* < 0.05). The S-LIr and Ir-S groups did not differ statistically (*p* > 0.05).

## 4. Discussion

Muscle activity is an important factor with regard to the quality of life and rehabilitation of patients undergoing head and neck radiotherapy [19]. In this context, the side effect that affects the masticatory muscles, resulting in trismus, can lead to difficulties in feeding, oral hygiene, speech, and weight loss, and the individual’s oral rehabilitation can also sometimes become impossible [20]. These difficulties are consequences of fibrosis of the temporomandibular joint, ligaments, and adjacent muscles, which lead to a loss of flexibility and consequently to a gradual reduction in mouth opening ability, which characterizes trismus [21]. In order to better understand the side effects of radiotherapy on healthy soft tissues, in this study, the influence of ionizing radiation on muscle contraction and on the skin was evaluated.

Few studies have evaluated the different clinical situations for irradiation in vivo studies with focus on skin and muscle. In the present study, the irradiation events were determined based on the previous study by Quinn et al. [17], which correlated rat ages with human ages. In the S-IIr group, the animals were subjected to surgical wounds and immediate irradiation, resulting in decreased muscle contractibility. In this case, the clinical situation is related to patients who have undergone buccal surgery and, in a short time, have submitted to radiotherapy. 

Previous studies reported that the tissue damage caused by ionizing radiation therapy can lead to inflammation of the skin and progress to ulcers or necrosis [22,23], and also that radiotherapy can delay the healing and renewal of tissues [23]. In addition, Najafi et al. [24] reported that radiation-induced DNA damage and cell death trigger an inflammatory response, due to signals that trigger the release of pro-inflammatory cytokines and an immunostimulatory response. 

Radiotherapy with single doses of 20–30 Gy is associated with changes in muscle morphology, while several fractional doses greater than 14 Gy cause irreversible apoptosis in endothelial cells [4]. Previous studies that used a single dose between 15 and 30 Gy reported the presence of muscle changes, both in fiber morphology and an increase in muscle fibrosis [4,25], as observed in this study. Clinical studies suggest that fibrosis results from the formation of excess fibrous connective tissue, resulting in decreased muscle motility [26] and atrophy of muscle fibers [27,28], corroborating with the results presented in this study that showed a smaller perimeter of muscle fibers in the irradiated groups compared to the control group, regardless of the radiotherapy time. It is noteworthy that when there was an association between the surgical procedure and irradiation, the perimeter of the fibers became considerably smaller, as in the S-IIr group, but this group did not exhibit lower values for EMG. This S group showed considerable changes in skin analysis when compared to the irradiated groups and the control group, which may be due to the radiosensitivity of involved tissue [23].

The S-LIr group aimed at simulating a clinical situation in which the surgical wound preceded the irradiation, and there was an interval between procedures of 4 weeks. McCal [29] recommended that surgeries should be performed at least 10 days before the beginning of radiotherapy, but for correct healing to occur, the ideal interval time is 21 days between the surgical procedure and the irradiation. This result is in agreement with the present study, since the S-LIr group did not show a statistical difference from the control group in regard to skin analyses. Regarding muscle analysis, this group exhibited lower values after irradiation when compared to the time after surgical wound, and this suggests the greater negative influence of radiotherapy in muscle activity as reported by Pauli et al. [30].

The worst EMG values were observed in the Ir-S group, both EMG 1 and EMG 2, and this result may be due to the prolonged effect of radiation, which promoted a decrease in muscle contractibility [31,32]. The Ir-S group represented the attempt to simulate the clinical situation of irradiated patients who, two years after the conclusion of cancer treatment, needed treatment involving surgical wounds. The irradiation of the animals in this group was performed four weeks before the surgical procedure. Based on these results, this time is short for indication of an elective surgery procedure. On the other hand, in the skin analysis, this group sometimes did not differ from the S-LIr and control groups, again probably due to the internal radiosensitivity of involved tissue [23].

Surgical and radiotherapeutic approaches are commonly used in the treatment of tumors located in the head and neck region; however, they are more strongly associated with severe cases of trismus [33]. This association was observed in our study, where the groups subjected to combined surgery and radiotherapy exhibited the worst levels of muscular contractility. By presenting experimental models of different clinical situations, our findings contribute to the ability to predict which individuals are more likely to develop soft tissue disorders, especially in muscles, at different stages of oncological treatment. This point is crucial for minimizing damage and sequelae and ensuring a better quality of life for patients, as also mentioned by Agarwal et al. [34]. By identifying signs early on, prophylactic therapies can be implemented, reducing the progression or even avoiding the course of the complication.

Starmer [35] suggests that radio-induced fibrosis is mitigated by swallowing exercises, preserving the muscles involved in stomatognathic functions. Hutcheson et al. [36] corroborate this idea by demonstrating, in a cohort of patients treated with combined radiotherapy and chemotherapy or radiotherapy alone, that long-term swallowing function outcomes were better among those who adhered to the exercises compared to those who did not adhere. Although it is possible to implement these exercises preventively, there is still a need to clarify the optimal timing to initiate them [37]. While this research can contribute to an early understanding of the side effects, it is not able to determine the appropriate timing for prophylactic intervention. Clinical studies involving patients irradiated at different stages of radiotherapy treatment and undergoing therapeutic exercises will be necessary to elucidate this aspect.

## 5. Conclusions

The current study showed that radiotherapy causes negative side effects in the surrounding soft tissue regardless of the application time. The results suggested that ionizing irradiation leads to a reduction in the perimeter and contractability of muscle fibers, as well as a lower amount of skin fiber renewal.

## Data Availability

Data will be made available on request from the corresponding author.
